# (*E*)-1-(2,5-Dichloro-3-thien­yl)-3-[4-(dimethyl­amino)phen­yl]prop-2-en-1-one

**DOI:** 10.1107/S1600536810014364

**Published:** 2010-04-24

**Authors:** Grzegorz Dutkiewicz, C. S. Chidan Kumar, H. S. Yathirajan, B. Narayana, Maciej Kubicki

**Affiliations:** a Department of Chemistry, Adam Mickiewicz University, Grunwaldzka 6, 60-780 Poznań, Poland; b Department of Studies in Chemistry, University of Mysore, Manasagangotri, Mysore 570 006, India; c Department of Studies in Chemistry, Mangalore University, Manasagangotri, Mangalagangotri 574 199, India

## Abstract

In the title compound, C_15_H_13_Cl_2_NOS, the benzene and thio­phene rings make a dihedral angle of 10.8 (1)°. The dimethyl­amino substituent and the α,β-unsaturated carbonyl group are almost coplanar with respect to the aromatic ring, forming dihedral angles of 4.73 (3)° and 5.0 (2)°, respectively. In the crystal structure, mol­ecules are connected into two-dimensional layers by weak C—H⋯Cl hydrogen bonds and C—Cl⋯O [Cl⋯O = 3.073 (2) Å] inter­actions. These layers are stacked with short C(meth­yl)–H⋯π contacts betweeen the layers.

## Related literature

For applications of chalcone derivatives, see: Indira *et al.* (2002 ▶); Sarojini *et al.* (2006 ▶); Tomar *et al.* (2007 ▶).
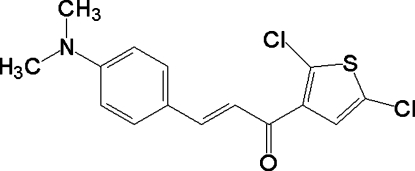


## Experimental

### 

#### Crystal data

C_15_H_13_Cl_2_NOS
*M* *_r_* = 326.22
Triclinic, 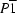

*a* = 7.2637 (9) Å
*b* = 8.1136 (9) Å
*c* = 13.478 (2) Å
α = 89.011 (9)°
β = 79.71 (1)°
γ = 73.07 (1)°
*V* = 747.2 (2) Å^3^
*Z* = 2
Mo *K*α radiation
μ = 0.57 mm^−1^
*T* = 295 K
0.6 × 0.3 × 0.3 mm

#### Data collection

Oxford Diffraction Xcalibur diffractometer with an Eos detector
Absorption correction: multi-scan (*CrysAlis PRO*; Oxford Diffraction, 2009 ▶) *T*_min_ = 0.785, *T*_max_ = 1.000
8710 measured reflections
3152 independent reflections
2403 reflections with *I* > 2σ(*I*)
*R*_int_ = 0.018

#### Refinement

*R*[*F* ^2^ > 2σ(*F* ^2^)] = 0.038
*wR*(*F* ^2^) = 0.109
*S* = 1.10
3152 reflections
184 parameters
H-atom parameters constrained
Δρ_max_ = 0.37 e Å^−3^
Δρ_min_ = −0.40 e Å^−3^

### 

Data collection: *CrysAlis PRO* (Oxford Diffraction, 2009 ▶); cell refinement: *CrysAlis PRO*; data reduction: *CrysAlis PRO*; program(s) used to solve structure: *SIR92* (Altomare *et al.*, 1993 ▶); program(s) used to refine structure: *SHELXL97* (Sheldrick, 2008 ▶); molecular graphics: *ORTEP-3* (Farrugia, 1997 ▶) and *Mercury* (Macrae *et al.*, 2008 ▶); software used to prepare material for publication: *Stereochemical Workstation Operation Manual* (Siemens, 1989 ▶) and *SHELXL97*.

## Supplementary Material

Crystal structure: contains datablocks I, global. DOI: 10.1107/S1600536810014364/im2193sup1.cif

Structure factors: contains datablocks I. DOI: 10.1107/S1600536810014364/im2193Isup2.hkl

Additional supplementary materials:  crystallographic information; 3D view; checkCIF report

## Figures and Tables

**Table 1:** Hydrogen-bond geometry (Å, °) *Cg* is the centroid of the phenyl ring.

*D*—H⋯*A*	*D*—H	H⋯*A*	*D*⋯*A*	*D*—H⋯*A*
C16—H16*B*⋯Cl5^i^	0.96	2.72	3.664 (2)	168
C16—H16*A*⋯*Cg*^ii^	0.96	3.01	3.899 (3)	155

Symmetry codes: (i) 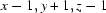; (ii) 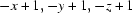.

**Fig. 1.**
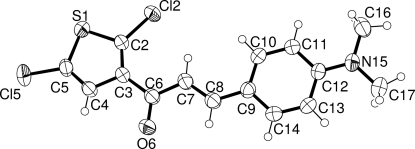
Molecular structure of (I), showing 50% probability displacement ellipsoids and the atom-numbering scheme.

**Fig. 2.**
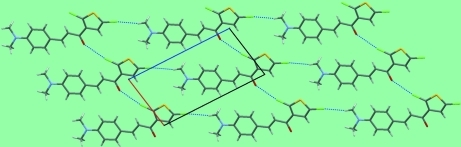
Crystal packing of (I) viewed along the b axis. C–H···Cl hydrogen bonds and C—Cl···O interactions are shown as dashed lines.

**Fig. 3.**
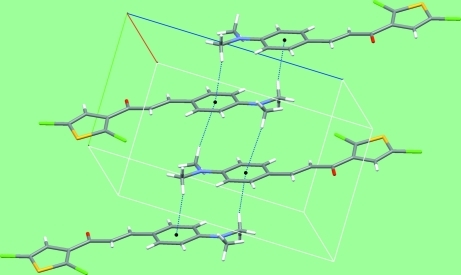
C–H···π interactions in the stack of molecules (I).

## supplementary crystallographic information

### Comment

Chalcones derivatives are known for their interesting pharmacological activities. Radical quenching properties of the phenolic groups present in many chalcones have raised interest in using the compounds themselves or chalcone rich plant extracts as drugs or food preservatives. Apart from being biologically important compounds, chalcone derivatives show non-linear optical properties with excellent blue light transmittance and good crystallizability (Indira *et al., *2002; Sarojini *et al.*, 2006). They provide a necessary configuration to show NLO property with two planar rings connected by a conjugated double bond. Synthesis and antimicrobial evaluation of new chalcones containing a 2,5-dichlorothiophene moiety is reported (Tomar *et al.*, 2007). Here, we report the synthesis and crystal structure of the new chalcone derivative, (2*E*)-1-(2,5-dichlorothiophen-3-yl)-3-(4-dimethylamino-phenyl)prop-2-en-1-one (I, Scheme 1) .

The molecule as a whole does not deviate significantly from planarity (Fig. 1). Dihedral angles between the constituent planar fragments are relatively small. The two ring planes of the phenyl and thiophene groups make a dihedral angle of 10.8 (1)°. The dimethylamino substituent and the α,β-unsaturated carbonyl moeity are inclined with respect to the phenyl ring plane by 4.73 (3)° and 5.0 (2)°, respectively. The bond lengths pattern within the C(=O)—C=C- fragment shows significant conjugation with shorter formal single bonds compared to formal double bonds that are longer than typical values.

In the crystal structure an intermolecular C–H···Cl hydrogen bond (H···Cl distance 2.72 Å, C–H···Cl angle 168°) and C—Cl···O interactions connect the molecules into approximately planar layers parallel to (101) (Fig. 2). The chlorine oxygen interaction also is almost linear (C2–Cl2···O6 angle of 167.6 (8)°) and relatively short (Cl2···O6 3.073 (2) Å). These layers are stacked on each other showing additional intermolecular C–H···π interactions with H16A···Cg distance of 3.01Å (Cg is the centroid of the phenyl ring).

### Experimental

1-(2,5-Dichlorothiophen-3-yl)ethanone (1.95 g, 0.01 mol) was mixed with 4-dimethylamino)-benzaldehyde (1.49 g, 0.01 mol) and dissolved in ethanol (30 ml). 3 ml of KOH (50%) was added to this solution. The reaction mixture was stirred for 6 hours. The resulting crude solid was filtered, washed successively with distilled water and finally recrystallized from ethanol (95%) to give the pure chalcone. Crystals suitable for x-ray diffraction studies were grown by slow evaporation of solution in toluene (M.P.: 358 K).

### Refinement

H atoms were placed in idealized positions and constrained to ride on their parent atoms, with C–H = 0.93 Å and U_iso_(H) = 1.2 U_eq_(C) for phenyl hydrogen and olefinic CH groups and with 0.96 Å and U_iso_(H) = 1.5 U_eq_(C) for CH_3_ groups.

### Crystal data

**Table:**

C_15_H_13_Cl_2_NOS	*Z* = 2
*M**_r_* = 326.22	*F*(000) = 336
Triclinic, *P*1	*D*_x_ = 1.450 Mg m^−^^3^
Hall symbol: -P 1	Mo *K*α radiation, λ = 0.71073 Å
*a* = 7.2637 (9) Å	Cell parameters from 5611 reflections
*b* = 8.1136 (9) Å	θ = 2.6–28.2°
*c* = 13.478 (2) Å	µ = 0.57 mm^−^^1^
α = 89.011 (9)°	*T* = 295 K
β = 79.71 (1)°	Block, yellow
γ = 73.07 (1)°	0.6 × 0.3 × 0.3 mm
*V* = 747.2 (2) Å^3^	

### Data collection

**Table:**

Oxford Diffraction Xcalibur diffractometer with an Eos detector	3152 independent reflections
Radiation source: Enhance (Mo) X-ray Source	2403 reflections with *I* > 2σ(*I*)
graphite	*R*_int_ = 0.018
Detector resolution: 16.1544 pixels mm^-1^	θ_max_ = 28.3°, θ_min_ = 2.6°
ω scan	*h* = −9→9
Absorption correction: multi-scan (*CrysAlis PRO*; Oxford Diffraction, 2009)	*k* = −10→10
*T*_min_ = 0.785, *T*_max_ = 1.000	*l* = −17→17
8710 measured reflections	

### Refinement

**Table:**

Refinement on *F*^2^	Primary atom site location: structure-invariant direct methods
Least-squares matrix: full	Secondary atom site location: difference Fourier map
*R*[*F*^2^ > 2σ(*F*^2^)] = 0.038	Hydrogen site location: inferred from neighbouring sites
*wR*(*F*^2^) = 0.109	H-atom parameters constrained
*S* = 1.10	*w* = 1/[σ^2^(*F*_o_^2^) + (0.0546*P*)^2^ + 0.1725*P*] where *P* = (*F*_o_^2^ + 2*F*_c_^2^)/3
3152 reflections	(Δ/σ)_max_ = 0.001
184 parameters	Δρ_max_ = 0.37 e Å^−^^3^
0 restraints	Δρ_min_ = −0.40 e Å^−^^3^

### Special details

**Table:**

Geometry. All s.u.'s (except the s.u. in the dihedral angle between two l.s. planes) are estimated using the full covariance matrix. The cell s.u.'s are taken into account individually in the estimation of s.u.'s in distances, angles and torsion angles; correlations between s.u.'s in cell parameters are only used when they are defined by crystal symmetry. An approximate (isotropic) treatment of cell s.u.'s is used for estimating s.u.'s involving l.s. planes.
Refinement. Refinement of *F*^2^ against ALL reflections. The weighted *R*-factor wR and goodness of fit *S* are based on *F*^2^, conventional *R*-factors *R* are based on *F*, with *F* set to zero for negative *F*^2^. The threshold expression of *F*^2^ > 2σ(*F*^2^) is used only for calculating *R*-factors(gt) etc. and is not relevant to the choice of reflections for refinement. *R*-factors based on *F*^2^ are statistically about twice as large as those based on *F*, and *R*- factors based on ALL data will be even larger.

### Fractional atomic coordinates and isotropic or equivalent isotropic displacement parameters (Å^2^)

**Table:**

	*x*	*y*	*z*	*U*_iso_*/*U*_eq_	
S1	0.67788 (8)	0.11979 (8)	1.11606 (4)	0.05238 (18)	
Cl2	0.49256 (8)	0.25700 (9)	0.94627 (4)	0.0620 (2)	
C2	0.6887 (3)	0.2294 (3)	1.00611 (14)	0.0407 (4)	
C3	0.8555 (3)	0.2780 (2)	0.98033 (14)	0.0383 (4)	
C4	0.9777 (3)	0.2234 (3)	1.05438 (15)	0.0431 (5)	
H4A	1.0980	0.2443	1.0507	0.052*	
Cl5	1.00237 (10)	0.05512 (9)	1.23148 (4)	0.0699 (2)	
C5	0.9014 (3)	0.1398 (3)	1.12908 (15)	0.0454 (5)	
C6	0.9255 (3)	0.3667 (3)	0.89011 (15)	0.0432 (5)	
O6	1.0970 (2)	0.3669 (2)	0.87491 (13)	0.0636 (5)	
C7	0.7927 (3)	0.4499 (3)	0.82226 (16)	0.0473 (5)	
H7A	0.6621	0.4519	0.8378	0.057*	
C8	0.8548 (3)	0.5232 (3)	0.73845 (16)	0.0456 (5)	
H8A	0.9845	0.5242	0.7283	0.055*	
C9	0.7452 (3)	0.6010 (2)	0.66156 (15)	0.0412 (4)	
C10	0.5490 (3)	0.6086 (3)	0.66361 (15)	0.0435 (5)	
H10A	0.4820	0.5665	0.7188	0.052*	
C11	0.4534 (3)	0.6765 (3)	0.58656 (15)	0.0435 (5)	
H11A	0.3233	0.6795	0.5908	0.052*	
C12	0.5478 (3)	0.7420 (2)	0.50101 (14)	0.0399 (4)	
C13	0.7440 (3)	0.7332 (3)	0.49862 (16)	0.0489 (5)	
H13A	0.8125	0.7735	0.4432	0.059*	
C14	0.8364 (3)	0.6662 (3)	0.57683 (16)	0.0500 (5)	
H14A	0.9662	0.6641	0.5731	0.060*	
N15	0.4527 (3)	0.8088 (2)	0.42422 (14)	0.0527 (5)	
C16	0.2469 (4)	0.8283 (3)	0.4313 (2)	0.0669 (7)	
H16A	0.2267	0.7165	0.4308	0.100*	
H16B	0.2015	0.8906	0.3749	0.100*	
H16C	0.1756	0.8906	0.4929	0.100*	
C17	0.5519 (4)	0.8642 (4)	0.33356 (17)	0.0657 (7)	
H17A	0.5962	0.9594	0.3497	0.098*	
H17B	0.4636	0.8996	0.2868	0.098*	
H17C	0.6623	0.7707	0.3037	0.098*	

### Atomic displacement parameters (Å^2^)

**Table:**

	*U*^11^	*U*^22^	*U*^33^	*U*^12^	*U*^13^	*U*^23^
S1	0.0501 (3)	0.0691 (4)	0.0431 (3)	−0.0253 (3)	−0.0097 (2)	0.0124 (3)
Cl2	0.0468 (3)	0.0961 (5)	0.0581 (4)	−0.0360 (3)	−0.0242 (3)	0.0189 (3)
C2	0.0371 (10)	0.0488 (11)	0.0378 (10)	−0.0123 (9)	−0.0117 (8)	0.0027 (8)
C3	0.0376 (10)	0.0399 (10)	0.0387 (10)	−0.0103 (8)	−0.0124 (8)	0.0024 (8)
C4	0.0383 (10)	0.0475 (12)	0.0464 (11)	−0.0125 (9)	−0.0160 (9)	0.0042 (9)
Cl5	0.0750 (4)	0.0881 (5)	0.0490 (3)	−0.0178 (3)	−0.0300 (3)	0.0209 (3)
C5	0.0479 (11)	0.0516 (12)	0.0372 (10)	−0.0104 (9)	−0.0162 (9)	0.0060 (9)
C6	0.0400 (10)	0.0458 (11)	0.0467 (11)	−0.0139 (9)	−0.0138 (9)	0.0059 (9)
O6	0.0461 (9)	0.0881 (12)	0.0681 (11)	−0.0317 (8)	−0.0226 (8)	0.0325 (9)
C7	0.0437 (11)	0.0525 (12)	0.0500 (12)	−0.0165 (10)	−0.0170 (9)	0.0138 (10)
C8	0.0436 (11)	0.0482 (12)	0.0490 (12)	−0.0165 (9)	−0.0139 (9)	0.0072 (9)
C9	0.0448 (11)	0.0398 (11)	0.0408 (10)	−0.0144 (9)	−0.0091 (8)	0.0057 (8)
C10	0.0447 (11)	0.0474 (12)	0.0389 (10)	−0.0165 (9)	−0.0046 (8)	0.0086 (9)
C11	0.0382 (10)	0.0496 (12)	0.0436 (11)	−0.0148 (9)	−0.0078 (8)	0.0083 (9)
C12	0.0451 (11)	0.0370 (10)	0.0379 (10)	−0.0119 (8)	−0.0085 (8)	0.0044 (8)
C13	0.0474 (12)	0.0568 (13)	0.0456 (12)	−0.0226 (10)	−0.0054 (9)	0.0145 (10)
C14	0.0404 (11)	0.0612 (14)	0.0528 (13)	−0.0217 (10)	−0.0098 (9)	0.0138 (10)
N15	0.0540 (11)	0.0618 (12)	0.0466 (10)	−0.0205 (9)	−0.0163 (8)	0.0205 (8)
C16	0.0601 (15)	0.0778 (17)	0.0707 (16)	−0.0228 (13)	−0.0302 (13)	0.0236 (13)
C17	0.0734 (17)	0.0800 (17)	0.0454 (13)	−0.0247 (14)	−0.0132 (12)	0.0191 (12)

### Geometric parameters (Å, °)

**Table:**

S1—C2	1.717 (2)	C10—C11	1.371 (3)
S1—C5	1.717 (2)	C10—H10A	0.9300
Cl2—C2	1.7175 (19)	C11—C12	1.411 (3)
C2—C3	1.366 (3)	C11—H11A	0.9300
C3—C4	1.433 (3)	C12—N15	1.364 (3)
C3—C6	1.489 (3)	C12—C13	1.401 (3)
C4—C5	1.333 (3)	C13—C14	1.371 (3)
C4—H4A	0.9300	C13—H13A	0.9300
Cl5—C5	1.718 (2)	C14—H14A	0.9300
C6—O6	1.226 (2)	N15—C17	1.439 (3)
C6—C7	1.462 (3)	N15—C16	1.443 (3)
C7—C8	1.335 (3)	C16—H16A	0.9600
C7—H7A	0.9300	C16—H16B	0.9600
C8—C9	1.444 (3)	C16—H16C	0.9600
C8—H8A	0.9300	C17—H17A	0.9600
C9—C14	1.391 (3)	C17—H17B	0.9600
C9—C10	1.403 (3)	C17—H17C	0.9600
			
C2—S1—C5	89.86 (10)	C10—C11—C12	121.54 (19)
C3—C2—S1	113.70 (14)	C10—C11—H11A	119.2
C3—C2—Cl2	130.86 (16)	C12—C11—H11A	119.2
S1—C2—Cl2	115.43 (11)	N15—C12—C13	121.88 (18)
C2—C3—C4	110.04 (18)	N15—C12—C11	121.47 (19)
C2—C3—C6	130.73 (17)	C13—C12—C11	116.65 (18)
C4—C3—C6	119.18 (17)	C14—C13—C12	120.89 (19)
C5—C4—C3	113.12 (18)	C14—C13—H13A	119.6
C5—C4—H4A	123.4	C12—C13—H13A	119.6
C3—C4—H4A	123.4	C13—C14—C9	123.04 (19)
C4—C5—S1	113.29 (15)	C13—C14—H14A	118.5
C4—C5—Cl5	127.12 (17)	C9—C14—H14A	118.5
S1—C5—Cl5	119.59 (13)	C12—N15—C17	121.73 (19)
O6—C6—C7	121.58 (19)	C12—N15—C16	121.05 (19)
O6—C6—C3	117.89 (17)	C17—N15—C16	117.22 (19)
C7—C6—C3	120.54 (17)	N15—C16—H16A	109.5
C8—C7—C6	121.49 (19)	N15—C16—H16B	109.5
C8—C7—H7A	119.3	H16A—C16—H16B	109.5
C6—C7—H7A	119.3	N15—C16—H16C	109.5
C7—C8—C9	127.96 (19)	H16A—C16—H16C	109.5
C7—C8—H8A	116.0	H16B—C16—H16C	109.5
C9—C8—H8A	116.0	N15—C17—H17A	109.5
C14—C9—C10	116.05 (18)	N15—C17—H17B	109.5
C14—C9—C8	120.06 (18)	H17A—C17—H17B	109.5
C10—C9—C8	123.80 (18)	N15—C17—H17C	109.5
C11—C10—C9	121.81 (18)	H17A—C17—H17C	109.5
C11—C10—H10A	119.1	H17B—C17—H17C	109.5
C9—C10—H10A	119.1		
			
C5—S1—C2—C3	0.36 (17)	C6—C7—C8—C9	176.1 (2)
C5—S1—C2—Cl2	179.45 (13)	C7—C8—C9—C14	−177.7 (2)
S1—C2—C3—C4	−0.3 (2)	C7—C8—C9—C10	−1.0 (4)
Cl2—C2—C3—C4	−179.25 (16)	C14—C9—C10—C11	0.0 (3)
S1—C2—C3—C6	176.89 (17)	C8—C9—C10—C11	−176.8 (2)
Cl2—C2—C3—C6	−2.0 (4)	C9—C10—C11—C12	0.1 (3)
C2—C3—C4—C5	0.1 (3)	C10—C11—C12—N15	179.72 (19)
C6—C3—C4—C5	−177.47 (18)	C10—C11—C12—C13	0.3 (3)
C3—C4—C5—S1	0.1 (2)	N15—C12—C13—C14	179.7 (2)
C3—C4—C5—Cl5	−179.88 (15)	C11—C12—C13—C14	−0.9 (3)
C2—S1—C5—C4	−0.28 (18)	C12—C13—C14—C9	1.0 (4)
C2—S1—C5—Cl5	179.74 (14)	C10—C9—C14—C13	−0.5 (3)
C2—C3—C6—O6	−166.3 (2)	C8—C9—C14—C13	176.4 (2)
C4—C3—C6—O6	10.7 (3)	C13—C12—N15—C17	4.0 (3)
C2—C3—C6—C7	13.5 (3)	C11—C12—N15—C17	−175.4 (2)
C4—C3—C6—C7	−169.47 (18)	C13—C12—N15—C16	−175.5 (2)
O6—C6—C7—C8	3.0 (3)	C11—C12—N15—C16	5.2 (3)
C3—C6—C7—C8	−176.8 (2)		

### Hydrogen-bond geometry (Å, °)

**Table:**

Cg is the centroid of the phenyl ring.

**Table:**

*D*—H···*A*	*D*—H	H···*A*	*D*···*A*	*D*—H···*A*
C16—H16B···Cl5^i^	0.96	2.72	3.664 (2)	168
C16—H16A···Cg^ii^	0.96	3.01	3.899 (3)	155

Symmetry codes: (i) *x*−1, *y*+1, *z*−1; (ii) −*x*+1, −*y*+1, −*z*+1.

## References

[bb1] Altomare, A., Cascarano, G., Giacovazzo, C. & Guagliardi, A. (1993). *J. Appl. Cryst* **26**, 343–350.

[bb2] Farrugia, L. J. (1997). *J. Appl. Cryst.* **30**, 565.

[bb3] Indira, J., Karat, P. P. & Sarojini, B. K. (2002). *J. Cryst. Growth*, **242**, 209–214.

[bb4] Macrae, C. F., Bruno, I. J., Chisholm, J. A., Edgington, P. R., McCabe, P., Pidcock, E., Rodriguez-Monge, L., Taylor, R., van de Streek, J. & Wood, P. A. (2008). *J. Appl. Cryst.* **41**, 466–470.

[bb5] Oxford Diffraction (2009). *CrysAlis PRO* Oxford Diffraction Ltd, Yarnton, England.

[bb6] Sarojini, B. K., Narayana, B., Ashalatha, B. V., Indira, J. & Lobo, K. G. (2006). *J. Cryst. Growth*, **295**, 54–59.

[bb7] Sheldrick, G. M. (2008). *Acta Cryst.* A**64**, 112–122. 10.1107/S0108767307043930 18156677

[bb8] Siemens (1989). *Stereochemical Workstation Operation Manual* Siemens Analytical X-ray Instruments Inc., Madison, Wisconsin, USA.

[bb9] Tomar, V., Bhattacharjee, G., Kamaluddin & Kumar, A. (2007). *Bioorg. Med. Chem. Lett.* **17**, 5321–5324. 10.1016/j.bmcl.2007.08.021 17719779

